# Anesthetic management of modified electroconvulsive therapy for a patient with coronary aneurysms: a case report

**DOI:** 10.1186/s40981-019-0298-y

**Published:** 2019-11-22

**Authors:** Riho Nakayama, Takuya Yoshida, Norihiko Obata, Satoshi Mizobuchi

**Affiliations:** Department of Anesthesiology, Kobe University Hospital, 7-5-2 Kusunoki-cho, Chuo-ku, Kobe city, Hyogo 650-0017 Japan

**Keywords:** Modified electroconvulsive therapy, Coronary artery aneurysm, Anesthetic management, ClearSight™ system

## Abstract

**Background:**

Modified electroconvulsive therapy (m-ECT) is utilized worldwide as an effective treatment for drug-resistant psychiatric disorders. However, during m-ECT, treatment of hypotension and hypertension in response to rapid hemodynamic changes is required. We used noninvasive continuous blood pressure monitoring system for continuous hemodynamic measurement during m-ECT.

**Case presentation:**

The patient was a 77-year-old man with depression complicated by coronary artery aneurysms (CAAs). We managed general anesthesia during m-ECT by using the ClearSight™ system (Edwards Lifesciences Corp, Irvine, CA, USA) for hemodynamic measurement. As a result, we performed a total of 10 m-ECTs. No rupture of CAAs or myocardial ischemia occurred and depressive symptoms improved.

**Conclusion:**

We successfully managed the anesthesia in m-ECT for a depressed patient with CAAs without complications by using the ClearSight™ system, which was used for the effective management of circulatory fluctuations.

## Background

Modified electroconvulsive therapy (m-ECT) is a biological treatment procedure involving a brief application of electrical stimulation to produce a generalized seizure [1]. During m-ECT, rapid changes in heart rate (HR) and blood pressure (BP) are reported to occur, so sufficient attention is required to manage fluctuations in circulatory dynamics [1].

We report a case of anesthetic management of m-ECT for a depressed patient with coronary artery aneurysms (CAAs) by using a noninvasive continuous blood pressure monitoring system (ClearSight™ system, Edwards Lifesciences Corp, Irvine, CA, USA), which makes possible a non-invasive, prompt response to changes of the circulatory dynamics.

## Case presentation

A 77-year-old man, 55 kg in weight and 165 cm in height, was scheduled to undergo m-ECT, due to a decreasing efficacy of drug treatment for depression over 9 years.

**Fig. 1:**
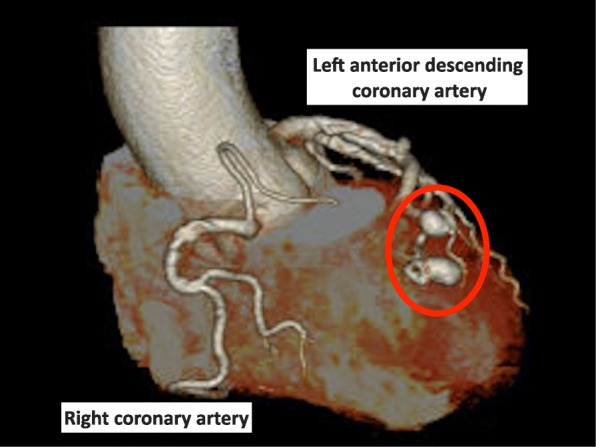
Preoperative coronary 3D-CT: Two coronary artery aneurysms are recognized in the periphery of the left anterior descending branch (red circle)

Seven years earlier, coronary computed tomography (CT) showed two CAAs with diameters of 7 × 8 mm and 6 × 11 mm at the distal first diagonal branch of the left coronary artery and right ventricular fistula formation (Fig. 1). The diameter of CAAs had no changes during the follow-up observation for 7 years. In adenosine myocardial load scintigraphy as a preoperative examination, neither myocardial ischemia nor infarction was detected. We surmised that the infarct range of the heart would be limited even if a thrombus was formed because the CAAs were located at the distal side of the left first diagonal branch. For that reason, we decided to prioritize m-ECT for depression rather than treatment for the CAAs.

**Fig. 2:**
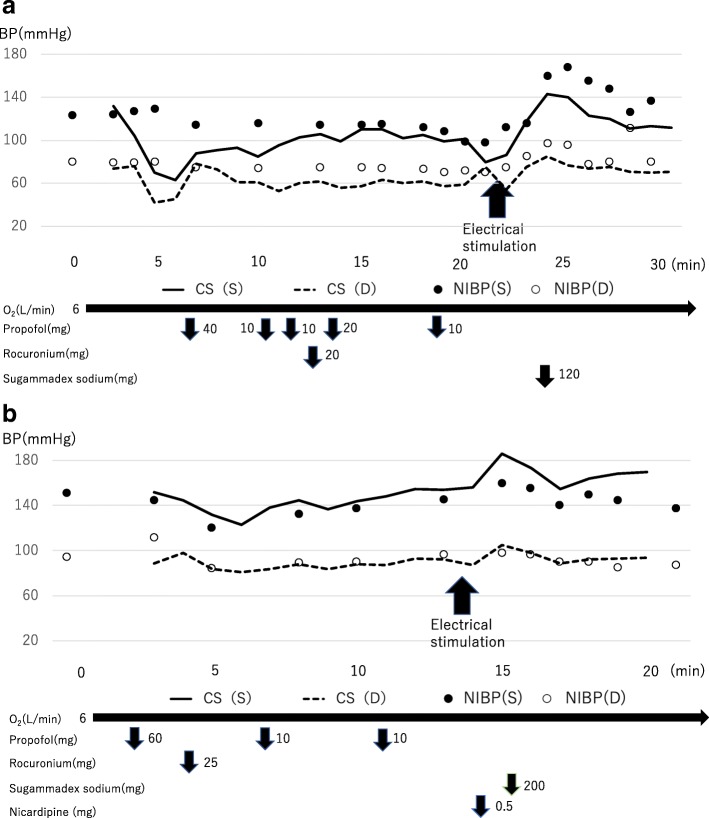
**a** The anesthetic chart of first m-ECT. **b** The anesthetic chart of second m-ECT. There was not much difference between NIBP and BP on the ClearSight™ system in both charts. CS (S): systolic blood pressure of the ClearSight™ system. CS (D): diastolic blood pressure of the ClearSight™ system. NIBP (S): systolic blood pressure of NIBP. NIBP (D): diastolic blood pressure of NIBP

Upon entering the operation room for the first m-ECT, the patient exhibited noninvasive blood pressure (NIBP) of 124/79 mmHg, and the BP on the ClearSight™ system was 132/74 mmHg (Fig. 2a). For setting the ClearSight™ system, we measured NIBP five times and confirmed that there was not much difference between NIBP and BP on the ClearSight™ system, and blood pressure was measured by this system throughout the procedure. General anesthesia was induced with propofol 1 mg kg^-1^, and after he lost consciousness, rocuronium 0.35 mg kg^-1^ was administered. Nine minutes after rocuronium administration, the psychiatrist performed electrical stimulation. As soon as the seizure started, HR increased from 83 beats min^-1^ to 102 beats min^-1^, and BP increased from 101/59 mmHg to 143/85 mmHg. The seizure duration was 51 s on the electroencephalogram. When the seizure stopped, the BP rapidly decreased to 120/75 mmHg without the use of any antihypertensive agent. After completing the seizure, sugammadex sodium 2 mg kg^-1^ was administered. When the patient regained consciousness, the BP was 112/71 mmHg. Neither asynergy nor pericardial effusion was detected by transthoracic echocardiography.

At the second m-ECT performed 4 days after the first procedure, when entering the operation room, NIBP was 144/111 mmHg, and the BP was 152/89 mmHg (Fig. 2b). General anesthesia was performed by referring to the first m-ECT. As soon as the seizure began after electrical stimulation, the BP increased from 155/93 mmHg to 186/105 mmHg. Therefore, nicardipine 0.01 mg kg^-1^ was administered, and BP decreased to 155/89 mmHg immediately. After completing the seizure, the BP was 164/92 mmHg. The seizure duration was 50 s on the electroencephalogram. We confirmed that there was no asynergy or pericardial effusion in the transthoracic echocardiography when he regained consciousness.

Both m-ECTs increased HR and BP. However, no decrease in HR or BP due to parasympathetic nervous stimulation or asystole was recognized. With reference to the first and second m-ECTs, we administered nicardipine hydrochloride prophylactically prior to the start of electrical stimulation every time after the third procedure. We performed a total of 10 m-ECTs, and the maximum NIBP in the all m-ECT procedures was 171/99 mmHg.

## Discussion

We present a case of successful anesthetic management in m-ECT for a patient with CAAs through prompt responses to changes in circulatory dynamics. As far as we know, this is the first report of m-ECT for a patient with CAA.

In most cases, CAA is asymptomatic, but the slow flow of blood on the irregular internal surface of the aneurysm wall predisposes to the formation of thrombi with subsequent embolization, resulting in myocardial ischemia and infarction and sudden death [2]. We found some reports of CAA rupture [3, 4], and there is also a report that hypertension triggered CAA rupture [5]. Therefore, we needed to control BP closely in m-ECT and decided to target systolic blood pressure below 180 mmHg.

It is known that rapid fluctuations in circulatory dynamics occur during m-ECT [1]. We have to be careful because, before or after the HR and BP increase due to sympathetic nerve stimulation accompanying the electrical stimulation, sometimes a parasympathetic response dominates, and there have been some case reports of bradycardia and 10 s of asystole [6, 7]. There have also been reports of cases in which asystole has occurred almost simultaneously with electrical stimulation [6]. Therefore, we thought that strict hemodynamic monitoring during m-ECT was needed. Because this case was complicated by CAAs, we decided to use the ClearSight™ system to see the hemodynamic change clearly during m-ECT, with the goal of not raising blood pressure excessively, to prevent CAA rupture.

The ClearSight™ system is a hemodynamic monitoring system that allows for real-time non-invasive BP measurements. Juri et al. reported the efficacy of the ClearSight™ system during cesarean section with accurate BP management [8]. Sumiyoshi et al. also reported that there is a significant relationship between mean arterial pressure and mean arterial pressure of the ClearSight™ system in patients with abdominal aortic aneurysm surgery [9]. Earle et al. reported that the ClearSight™ system was useful in m-ECT for a patient with abdominal aortic aneurysms [10]. In Earle’s report, the waveform of the ClearSight™ system disappeared for approximately 20 s due to electrical stimulation in m-ECT. However, when we used the ClearSight™ system, the arterial pressure waveform did not disappear during m-ECT, so we could monitor BP continuously and manage patient safety.

The first and second m-ECTs with the ClearSight™ system showed that electrical stimulation increased HR and BP but did not cause asystole and that prophylactic nicardipine administration did not cause excessive BP reduction.

We performed a total of 10 m-ECTs. No rupture of CAAs or myocardial ischemia occurred, and depressive symptoms improved through this series of m-ECTs.

In conclusion, we successfully managed the anesthesia in m-ECT for a depressed patient with CAAs by using the ClearSight™ system, which was used for the effective management of BP fluctuations during m-ECT.

## Data Availability

The data in this case report are available from the corresponding author on reasonable request.

## Abbreviations

BIS: Bispectral index
BP: Blood pressure
CAA: Coronary artery aneurysm
CT: Computed tomography
HR: Heart rate
m-ECT: Modified electroconvulsive therapy
NIBPq: Noninvasive blood pressure

## References

[1] Kerner N, Prudic J (2014). Current electroconvulsive therapy practice and research in the geriatric population. Neuropsychiatry (London)..

[2] Abou Sherif S, Ozden Tok O, Taskoylu O, Goktekin O, Kilic ID (2017). Coronary artery aneurysms: a review of the epidemiology, pathophysiology, diagnosis, and treatment. Front Cardiovasc Med..

[3] Kondo T, Takahashi M, Nakagawa K, Kuse A, Morichika M, Sakurada M (2015). Rupture of massive coronary artery aneurysm resulting in cardiac tamponade. Leg Med (Tokyo)..

[4] Kimura S, Miyamoto K, Ueno Y (2006). Cardiac tamponade due to spontaneous rupture of large coronary artery aneurysm. Asian Cardiovasc Thorac Ann..

[5] Iwasawa Y, Kitamura Y, Higuma K, Ono F, Imoto K, Kimura K (2007). Cardiac tamponade due to rupture of coronary artery fistulas with a giant aneurysm containing a free floating ball thrombus: a case report. J Cardiol..

[6] Marnie R, Geoffery L (2004). Asystole during successive electroconvulsive therapy sessions: a report of two cases. Journal of Clinical Anesthesia..

[7] Ethan O, Matthew F, Justin P (2018). Vagally mediated postictal asystole during electroconvulsive therapy. Journal of ECT..

[8] Juri T, Suehiro K, Kimura A, Mukai A, Tanaka K, Yamada T (2018). Impact of non-invasive continuous blood pressure monitoring on maternal hypotension during cesarean delivery: a randomized-controlled study. J Anesth..

[9] Sumiyoshi M, Maeda T, Miyazaki E, Hotta N, Sato H, Hamaguchi E (2019). Accuracy of the ClearSight system in patients undergoing abdominal aortic aneurysm surgery. J Anesth..

[10] Earle R, Vaghadia H, Sawka A (2015). Novel use of the Nexfin HD monitor for hemodynamic management during electroconvulsive therapy in a patient with an unrepaired abdominal aortic aneurysm. Can J Anaesth..

